# Supplementary material for: HIVprotI: an integrated web based platform for prediction and design of HIV proteins inhibitors
Source: J Cheminform. 2018 Mar 9;10:12. doi: 10.1186/s13321-018-0266-y (PMC5845081; doi:10.1186/s13321-018-0266-y)
Supplement: Supplementary file 2 — Additional file 2. Source code of HIVProtI web server. [file 13321_2018_266_MOESM2_ESM.zip › HIVprotI_Source-code/hivprotI/weka-3-6-6/doc/weka/gui/sql/event/ResultChangedEvent.html]

ResultChangedEvent


---


|  |  |  |
| --- | --- | --- |
| **Overview** \| **Package** \| **Class** \| **Tree** \| **Deprecated** \| **Index** \| **Help** \| **Weka's home** | |  |
| **PREV CLASS**   **NEXT CLASS** | **FRAMES**    **NO FRAMES**     **All Classes** |  |
| SUMMARY: NESTED \| FIELD \| CONSTR \| METHOD | DETAIL: FIELD \| CONSTR \| METHOD |  |


---


## weka.gui.sql.event Class ResultChangedEvent

```
java.lang.Object
  java.util.EventObject
      weka.gui.sql.event.ResultChangedEvent
```

**All Implemented Interfaces:**: java.io.Serializable

---

``` public class ResultChangedEvent extends java.util.EventObject ```

An event that is generated when a different Result is activated in the
ResultPanel.

**Version:**
:   $Revision: 1.2 $

**Author:**
:   FracPete (fracpete at waikato dot ac dot nz)

**See Also:**: `ResultChangedListener`, Serialized Form

---

**Constructor Summary**

`ResultChangedEvent(java.lang.Object source,
java.lang.String url,
java.lang.String user,
java.lang.String pw,
java.lang.String query)`
  
          constructs the event


| **Method Summary** | |
| --- | --- |
| `java.lang.String` | `getPassword()`             returns the password that produced the table model |
| `java.lang.String` | `getQuery()`             returns the query that was executed |
| `java.lang.String` | `getURL()`             returns the database URL that produced the table model |
| `java.lang.String` | `getUser()`             returns the user that produced the table model |
| `java.lang.String` | `toString()`             returns the event in a string representation |

**Methods inherited from class java.util.EventObject**

`getSource`

**Methods inherited from class java.lang.Object**

`equals, getClass, hashCode, notify, notifyAll, wait, wait, wait`

**Constructor Detail**

### ResultChangedEvent

```
public ResultChangedEvent(java.lang.Object source,
                          java.lang.String url,
                          java.lang.String user,
                          java.lang.String pw,
                          java.lang.String query)
```

:   constructs the event

    **Parameters:**: `source` - the source that generated this event: `url` - the current database url: `user` - the current user: `pw` - the current password: `query` - the current query


**Method Detail**

### getURL

```
public java.lang.String getURL()
```

:   returns the database URL that produced the table model

---


### getUser

```
public java.lang.String getUser()
```

:   returns the user that produced the table model

---


### getPassword

```
public java.lang.String getPassword()
```

:   returns the password that produced the table model

---


### getQuery

```
public java.lang.String getQuery()
```

:   returns the query that was executed

---


### toString

```
public java.lang.String toString()
```

:   returns the event in a string representation

    :   **Overrides:**: `toString` in class `java.util.EventObject`
    :   **Returns:**: the event in a string representation


---


|  |  |  |
| --- | --- | --- |
| **Overview** \| **Package** \| **Class** \| **Tree** \| **Deprecated** \| **Index** \| **Help** \| **Weka's home** | |  |
| **PREV CLASS**   **NEXT CLASS** | **FRAMES**    **NO FRAMES**     **All Classes** |  |
| SUMMARY: NESTED \| FIELD \| CONSTR \| METHOD | DETAIL: FIELD \| CONSTR \| METHOD |  |


---
